# The role of β-catenin in cardiac diseases

**DOI:** 10.3389/fphar.2023.1157043

**Published:** 2023-03-22

**Authors:** Beibei Ni, Meijuan Sun, Jun Zhao, Jiao Wang, Zhanqi Cao

**Affiliations:** Department of Pharmacy, The Affiliated Hospital of Qingdao University, Qingdao, China

**Keywords:** Wnt pathway, β-catenin, cardiac diseases, inhibitors, strategies

## Abstract

The Wnt/β-catenin signaling pathway is a classical Wnt pathway that regulates the stability and nuclear localization of β-catenin and plays an important role in adult heart development and cardiac tissue homeostasis. In recent years, an increasing number of researchers have implicated the dysregulation of this signaling pathway in a variety of cardiac diseases, such as myocardial infarction, arrhythmias, arrhythmogenic cardiomyopathy, diabetic cardiomyopathies, and myocardial hypertrophy. The morbidity and mortality of cardiac diseases are increasing, which brings great challenges to clinical treatment and seriously affects patient health. Thus, understanding the biological roles of the Wnt/β-catenin pathway in these diseases may be essential for cardiac disease treatment and diagnosis to improve patient quality of life. In this review, we summarize current research on the roles of β-catenin in human cardiac diseases and potential inhibitors of Wnt/β-catenin, which may provide new strategies for cardiac disease therapies.

## 1 Introduction

Wnt signaling pathways are highly conserved pathways that are well known for their regulatory roles in embryonic development, tissue regeneration and adult tissue homeostasis (Hayat et al., 2022). Wnt signaling pathways are categorized into two groups: canonical and non-canonical pathways. The canonical pathway is β-catenin dependent and is known as the Wnt/β-catenin signaling pathway. The non-canonical Wnt pathway is β-catenin-independent and mainly includes the non-canonical Wnt planar cell polarity (Wnt/PCP) and Wnt/Ca^2+^ pathways. Canonical Wnt/β-catenin is primarily involved in the regulation of cell fate decisions and proliferation during embryonic development and tissue homeostasis in adults, while non-canonical pathways mainly regulate the polarity, mobility and migration of the cell (Majidinia et al., 2018; Li et al., 2022). Cardiovascular diseases are still a serious threat to human life and health. The presence of cardiac diseases can increase the risk of sudden death (Bagnall et al., 2020). Although the medical strategies are gradually improving, the treatment of these diseases still faces severe challenges. In recent years, many studies have shown that activation of the Wnt/β-catenin signaling pathway could further participate in the healing and repair of myocardial infarction, myocardial hypertrophy, myocardial fibrosis, ventricular remodeling, heart failure and other pathophysiological processes (Umbarkar et al., 2021; Zhang Q. et al., 2022; Zhang Y. et al., 2022). This review aims to summarize the mechanism of the Wnt/β-catenin pathway in cardiac diseases and potential inhibitors of Wnt/β-catenin discovered in recent years, which may provide new strategies for cardiac disease therapies.

## 2 The Wnt/β-catenin signaling pathway

The Wnt/β-catenin pathway is composed of four segments: extracellular ligand protein Wnts, receptors on the cell membrane, the cytoplasmic segment for signal transduction, and the nuclear segment for transcriptional regulation. The main components of the Wnt/β-catenin signaling pathway include Wnts, low-density lipoprotein receptor-related protein receptors 5/6 (LRP5/6), frizzled (Fzd), dishevelled (Dvl), GSK-3β, Casein kinase 1α (CK1α), Axin, Adenomatosis Escherichia coli (APC), Protein phosphatase 2A (PP2A), E3-ubiquitin ligase β-TrCP, β-catenin, and T-cell factor/lymphoid enhancer factor (TCF/LEF). Wnts are secreted lipoglycoproteins. Nineteen Wnt proteins have been identified in mammals. Among them, Wnt1, Wnt3a, Wnt7a, Wnt7b, Wnt 8 and Wnt 10a act as ligands to activate the canonical Wnt/β-catenin pathway (Balatskyi et al., 2023). Wnts require glycosylation and palmitoylation to acquire the ability to be secreted, and these factors act in both an autocrine and paracrine manner. Fzd are class F G protein-coupled receptors and act as receptors of Wnts. LRP5/6 are coreceptors of Wnts and are located on the plasma membrane. Dvl is the cytoplasmic protein that acts as a key signal transducer from receptors to downstream effectors. In addition, another role of Dvl has been discovered. Dvl functions as an adaptor to recruit the negative regulators zinc and RING finger 3 (ZNRF3)/RING finger protein 43 (RNF43) to promote Wnt degradation and prevent pathway overactivity (Jiang et al., 2015). The destruction complex, which is composed of GSK-3β, CK1α, Axin, APC, PP2A and β-TrCP, is responsible for β-catenin proteasomal degradation in the absence of Wnt stimulation (Bagchi and MacDougald, 2021). β-catenin is a key downstream effector of the Wnt/β-catenin pathway. The Wnt/β-catenin pathway leads to the accumulation of β-catenin in the cytoplasm, which allows β-catenin nuclear translocation and subsequently induces the transcription of target genes *via* TCF/LEF transcription factors (Liu et al., 2022). In addition, β-catenin can participate in the formation of the E-cadherin/β-catenin complex to mediate the linkage of cadherins and cell conjugation (Xu et al., 2016). Interestingly, there are two functional pools of β-catenin, one associated with transcription and one associated with adhesion, and these pools are not completely separated (van der Wal and van Amerongen, 2020).

In the absence of Wnt signals, a destruction complex is formed. AXIN and APC provide a scaffold for the recruitment of GSK3β, CK1α, and β-catenin (Stamos and Weis, 2013). Once bound, GSK-3β phosphorylates β-catenin at Thr41, Ser33, and Ser37, and CK-1α phosphorylates β-catenin at Ser45. Then, phosphorylated β-catenin detaches from the complex and undergoes ubiquitin degradation *via* β-TrCP. As a result, β-catenin is maintained at a low level in the cytoplasm and nucleus. In the presence of Wnt signals, the Wnt protein binds to the extracellular N-terminal domain under the synergistic action of LRP5/6. During this process, FZD is homodimerized, and FZD-LRP5/6 is heterodimerized. Subsequently, Dvl proteins in the cytoplasm are recruited to the cell membrane and further oligomerized. DvL contains three main domains: the N-terminal DIX domain, central PDZ domain and C-terminal DEP domain. Dvl oligomerization recruits AXIN. Therefore, AXIN-bound GSK3β and CK1α can get close to LRP5/6 and phosphorylate it. Phosphorylated LRP5/6 can provide an AXIN binding site (Liu et al., 2022). Dvl binds to phosphorylated LRP5/6 Axin and inactivates the destruction complex. Accumulated β-catenin in the cytoplasm translocates into the nucleus and binds to the TCF/LEF cofactor to induce the transcription of target genes, such as cyclin D1, MMPs and c-Myc (Yu et al., 2021).

## 3 The role of β-catenin in cardiac development and function

The Wnt/β-catenin pathway is an important regulator of cardiac development and growth, and its activity in healthy adult hearts is low. Even so, this state of low activity is essential for maintaining normal heart function. Acute activation of the Wnt/β-catenin pathway is thought to play a cardioprotective role after infarction through the upregulation of prosurvival genes and metabolic reprogramming (Wang Q. et al., 2021). However, long-term high Wnt/β-catenin pathway activity may lead to prefibrosis and hypertrophy in the adult heart (Wang Q. et al., 2021). Wnt/β-catenin coordinates normal heart formation through spatiotemporal activation or inhibition (Piven and Winata, 2017). A previous study identified ectopic heart formation following conditional β-catenin inactivation in the final endoderm of mouse embryos, revealing the inhibitory role of Wnt/β-catenin signaling in vertebrate heart specification (Lickert et al., 2002). However, studies of zebrafish and mouse embryos and mouse and human embryonic stem cells (hESCs) have established that Wnt/β-catenin signaling induces differentiation early in vertebrate heart development but inhibits differentiation later (Ozhan and Weidinger, 2015). In addition, β-catenin was reported to promote cardiomyocyte proliferation in mice and zebrafish (Tseng et al., 2006; Bertozzi et al., 2022).

β-Catenin was confirmed to affect the development and function of the heart by participating in the metabolism of cardiomyocytes (Balatskyi et al., 2020; Balatskyi et al., 2021). A recent review showed that β-catenin could affect perinatal cardiometabolic maturation by altering glucose and fatty acid utilization (Balatskyi et al., 2023). β-catenin ablation caused mitochondrial insufficiency in perinatal cardiomyocytes, and hearts with heterozygous β-catenin ablation failed to increase mitochondrial numbers in response to exercise training (Balatskyi et al., 2020; Balatskyi et al., 2021). Additionally, the ablation of emerin, an inhibitor of β-catenin nuclear import, leads to heart dysfunction and aggravates cardiac remodeling following pressure overload (Stubenvoll et al., 2015). During the developmental stage of the heart, β-catenin ablation could lead to metabolic failure and significantly inhibit glycolysis in perinatal cardiomyocytes (Balatskyi et al., 2023). In the prenatal stage, oxygen partial pressure is relatively low, the heart is dependent on hypoxia-inducible factor 1α (HIF-1α) and rapamycin-driven metabolic patterns of mammalian/mechanical targets, and energy production occurs primarily through glycolysis (Liang and Ward, 2006). Synergies of β-catenin with HIF-1α and other TFs are thought to be required for metabolic patterning of the developing heart, which promotes cardiomyocyte proliferation in dense myocardium (Balatskyi et al., 2023). In the adult heart, β-catenin activation appears to result in a similar cardiac response to utilize more glucose (Balatskyi et al., 2023). Moreover, the regulatory effect of β-catenin on cardiomyocyte proliferation may also be related to the Hippo pathway (Heallen et al., 2011). In mouse embryonic hearts, Hippo pathway inactivation results in heart enlargement, resulting in increased cardiomyocyte quantity and cardiomyocyte proliferation (Heallen et al., 2011; Di Sante et al., 2023). The expression of β-catenin was upregulated in this heart, and deleting one copy of β-catenin could rescue the cardiac overgrowth phenotype. Overall, the Wnt/β-catenin pathway plays an irreplaceable role in cardiac development and function. Dysregulation of the Wnt/β-catenin pathway has catastrophic effects on the heart. The phenotypes in cardiac development and function caused by Wnt/β-catenin pathway-related genomic knockout are shown in Table 1.

**TABLE 1:** Sevaral phenotypes associated with cardiac development and function caused by Wnt/β-catenin pathway-related genomic knockout.

Genes/Proteins	Models	Specific cell	Phenotypes	Ref
*Ctnnb1*/β-catenin	neonatal mouse	cardiomyocyte	1. Died within 7 days	Balatskyi et al. (2021)
			2. Decreased genes associated with lipid catabolism in the heart	
			3. Decreased genes associated with anaerobic glycolysis in the heart	
			4. Mitochondrial dysfunction in the heart	
*Ctnnb1*/β-catenin	adult mouse	cardiomyocyte	1. Reduced cardiomyocytes size	Balatskyi et al. (2020)
			2. Decreased heart rate	
			3. Decreased expression of genes associated with hypertrophic response	
*Ctnnb1*/β-catenin	Fetal mouse	cardiomyocyte	1. Fetal survival rate decreased	Ye et al. (2015)
			2. Smaller hearts and thinner ventricular walls	
			3. Decreased mRNA levels of *Cyclin D2* in fetal hearts at embryonic Day 13.5 and 17.5	
*Ctnnb1*/β-catenin	adolescent mouse	cardiomyocyte	1. Decreased expression of *c-Myc* and *c-Fos*	Chen et al. (2006)
			2. Decreased hypertrophic response to thoracic aortic constriction (TAC)	
*Ctnnb1*/β-catenin	mouse embryo	embryonic stem cell	1. Ectoderm developmental disorder	Haegel et al. (1995)
			2. Mesoderm was absent	
*Ppp2ca*/PP2A	adult mouse	cardiomyocyte	Developed cardiomyocyte hypertrophy, fibrosis and heart failure	Li et al. (2016)
*Gsk3b/*GSK-3β	adult mouse	cardiomyocyte	Anterior wall hypertrophy	Stachowski-Doll et al. (2022)

## 4 β-catenin and cardiac diseases

### 4.1 β-catenin and myocardial infarction

At present, myocardial infarction (MI) is a cardiac disease with high morbidity and mortality. Acute MI is an event associated with myocardial necrosis caused by ischemia and hypoxia in the coronary arteries. MI is usually accompanied by oxidative stress, inflammatory responses and the transformation of cardiac fibroblasts to cardiac myofibroblasts (Frangogiannis, 2015). The Wnt/β-catenin pathway is silent in normal hearts but activated in human infarcted hearts (Wang Q. et al., 2021). It was reported that this pathway was not activated during the first 24 h after infarction but was gradually activated after 24 h, peaked at 7 days, and disappeared 3 weeks after infarction (Aisagbonhi et al., 2011). The Wnt/β-catenin pathway could be involved in multiple pathophysiological processes related to MI (Daskalopoulos and Blankesteijn, 2021).

#### 4.1.1 β-catenin and oxidative stress in MI

Changes in the defense mechanism against oxygen free radicals during myocardial infarction and myocardial reperfusion can cause oxidative stress damage (Chen, 2021). Wnt/β-catenin pathway activation was observed in an *in vitro* model of oxidation-damaged cardiomyocytes. Reactive oxygen species (ROS) can activate phosphatidylinositol 3 kinase (PI3K)/protein kinase B (AKT) by inhibiting phosphatase and tensin homolog deleted from chromosome (PTEN). Phosphorylated AKT was shown to activate the Wnt/β-catenin pathway by phosphorylating GSK-3β (Vallée and Lecarpentier, 2018). In addition, oxidative stress can activate the Wnt/β-catenin pathway by inhibiting the binding of nucleoredoxin (a thioredoxin family protein) to the Dvl PDZ domain. This binding of nucleoredoxin and PDZ blocks the downstream transduction of Wnt proteins transduced by Dvl and the stabilization of cytoplasmic β-catenin (Funato et al., 2006). Activation of the nuclear β-catenin/c-Myc axis promoted DNA damage and p53-mediated apoptosis induced by oxidative stress (Liu et al., 2017). Inhibition of the Wnt/β-catenin pathway attenuated DNA damage and apoptosis induced by oxidative stress (Qiu et al., 2017). Furthermore, the transfection of β-catenin plasmids in cardiomyocytes could increase the expression of apoptosis-related proteins, including Bax, cytochrome c and caspase-3 (Lin et al., 2017). In the MI model of β-catenin-knockout mice, the levels of proapoptotic active caspase-3 and Bax were significantly lower and antiapoptotic Bcl-2 levels were increased (Wo et al., 2016). These results suggest that β-catenin can promote oxidative stress injury.

**FIGURE 1:**
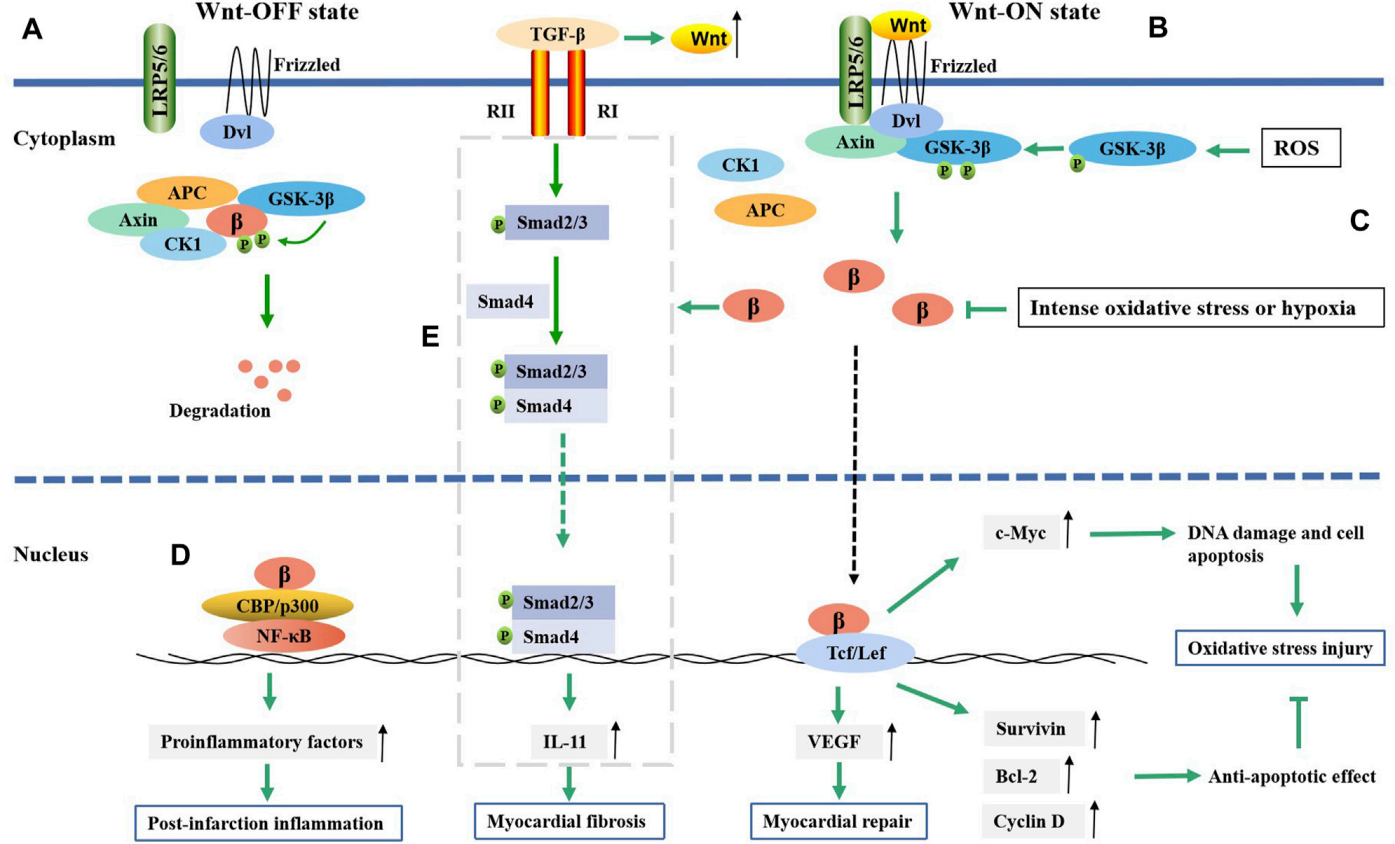
The regulatory mechanism of Wnt/β-catenin pathway in myocardial infarction. **(A)** In the absence of Wnt ligands, Wnt/β-catenin pathway is suppressed. **(B)** In the presence of Wnt ligands, Wnt/β-catenin pathway is activated. Then isolated β-catenin can translate into nucleus and bind to TCF/LEF, which will result in gene transcription such as Bcl-2, survivin, cyclin D1, c-Myc, *etc.* The exression of Bcl-2, survivin and cyclin D1 could inhibit oxidative stress injury, while c-Myc exhibits an opposite effect. **(C)** ROS promotes the phosphorylation of GSK-3β and then active the Wnt/β-catenin pathway, while intense oxidative stress or hypoxia can decrease the level of β-catenin. **(D)** β-catenin promotes NF-κB target genes expression through forming complex with NF-κB, which then promote post-infarction inflammation. **(E)** β-catenin can promote the TGF-β-mediated IL-11 expression in some way, which then induces the fibroblast-to-myofibroblast transition; β, β-catenin.

However, an inconsistent conclusion also exists. Some studies have confirmed that β-catenin/TCF-mediated transcription can play an antiapoptotic role after MI (Al-Salam et al., 2020; Zheng et al., 2021). After injecting active β-catenin into the infarct border zone of the mouse MI model, the infarct size was reduced. This effect occurred through increased nuclear translocation of β-catenin, enhanced transcriptional activity, and enhanced expression of survivin, Bcl-2, cyclin D, and cyclin E2, which ultimately led to an antiapoptotic effect on cardiomyocytes and cardiac fibroblasts (Zheng et al., 2021). This phenomenon was also confirmed in the hypoxia model. In a state of intense oxidative stress or hypoxia, the amount of β-catenin decreases, and the activity of the Wnt/β-catenin pathway is impaired. After scavenging accumulated ROS, the Wnt/β-catenin pathway is reactivated to produce an antiapoptotic effect (Zhou et al., 2017; Zhu and Lu, 2019). These inconsistent results may be due, on the one hand, to the use of different animal and cell models. On the other hand, activation of the Wnt/β-catenin pathway after MI is time-, space- and cell type-specific (Aisagbonhi et al., 2011). In short, oxidative stress and β-catenin activity may interact with each other and undergo dynamic changes. β-Catenin plays an important role in DNA damage and cell necrosis induced by oxidative stress after MI (Figure 1).

#### 4.1.2 β-catenin and inflammation in MI

The *postmortem* inflammatory response is important for postinfarction repair, but excessive inflammation is an important reason for myocardial remodeling and heart failure. Apoptosis and necrosis in myocardial cells after MI are the main factors that activate the inflammatory response. Inflammatory cell infiltration is usually observed in the infarct area (Chen and Frangogiannis, 2021). Macrophages in the infarct area react first, engulf necrotic myocardial cells, and secrete cytokines and chemokines. The Wnt/β-catenin pathway is inactive in normal cardiac macrophages. Increased levels of dissociative β-catenin in the cytoplasm were observed in cardiac macrophages, especially Ly6C + proinflammatory macrophages post MI (Huang et al., 2018). Activated β-catenin upregulated the expression of IL-1b, IL-6, TNF-a and IL-23 p19, which could then promote the macrophage-mediated inflammatory response (Huang et al., 2018). β-catenin might play a role in regulating MI inflammation through its interaction with NF-κB. Both the Wnt/β-catenin pathway and NF-κB were activated after MI (Song et al., 2022). NF-κB is a major transcription factor in the inflammatory response that can promote the expression of proinflammatory factors and enzymes. β-catenin promotes NF-κB target gene expression *via* the binding of the β-catenin-TCF/LEF complex to the promoters of NF-κB target genes (Figure 1). Overexpression of β-catenin in cardiomyocytes increased the expression of the inflammatory markers TNF-α, p-NF-κB and IL-8 and promoted the accumulation of NF-κB in the nucleus, suggesting that β-catenin promoted the occurrence of inflammation by activating NF-κB (Lin et al., 2016). Despite the complex relationship between β-catenin and inflammation, β-catenin may be a potential regulatory target for postinfarction inflammation.

#### 4.1.3 β-catenin and myocardial repair after MI

The restorative fibrosis response to injury at the early stage after MI is an important step in myocardial repair. Necrotic cardiomyocytes are replaced by proliferating fibroblasts and extracellular matrix (ECM), which effectively prevents cardiac rupture. β-Catenin plays an important role in myocardial fibrosis. It was found that overexpression of β-catenin could upregulate various fibrosis markers in fibroblasts (Liu et al., 2021). Moreover, Bing Zou et al. (Zou et al., 2021) showed that activation of the Wnt/β-catenin pathway was related to improving cardiac function in hypoxia cell models and MI rats. High β-catenin expression could promote the epithelial mesenchymal transition (EMT), which might be an important source of angiogenesis and muscle fiber cells and play an important role in tissue repair (Moheimani et al., 2015). The expression of Wnt1 and β-catenin in epicardial cells and fibroblasts increased within 2 weeks after acute ischemic heart injury. EMT in epicardial cells promoted fibroblast proliferation and increased the expression of profibrotic genes (Col1, Col3 and ET-1) (Duan et al., 2012). The interruption of the Wnt/β-catenin pathway in epicardial and fibroblast cells could lead to EMT deficiency and decrease fibroblast proliferation in mice, resulting in epicardial dilatation and impaired cardiac function (Duan et al., 2012).

It was also reported that β-catenin overexpression could not only induce fibroblast DNA replication and increase the number of fibroblasts but also induce the differentiation of fibroblasts into myofibroblasts (Dong et al., 2018). Several studies have indicated that β-catenin promotes fibrosis in myofibroblasts through transforming growth factor-β1 (TGF-β) (Carthy et al., 2011; Hao et al., 2016; Zhu et al., 2021). TGF-β was reported to be a key protein regulating fibroblast-to-myofibroblast differentiation (Tarbit et al., 2019). TGF-β is activated by mechanical damage and stimulation of the cellular environment after MI (Hanna and Frangogiannis, 2019). The Wnt/β-catenin pathway promotes TGF-β-mediated fibroblast-to-myofibroblast transition by enhancing interleukin-11 (IL-11) production at the transcriptional level (Działo et al., 2021). The expression of IL-11 is associated with activation of the TGF-β-mediated Smad signaling pathway (Yin et al., 2019). IL-11 is a downstream effector of TGFβ1 in fibroblasts and is required for extracellular-regulated kinase (ERK)-dependent autocrine signaling to drive fibrogenic protein synthesis (Schafer et al., 2017) (Figure 1). In addition, β-catenin could promote the differentiation of cardiac fibroblasts into myofibroblasts by increasing vascular endothelial growth factor (VEGF) expression (Li et al., 2021). Multiple studies have demonstrated that early inhibition of the Wnt/β-catenin pathway after MI can reduce myocardial fibrosis, reduce infarct size, and improve myocardial remodeling and cardiac function, indicating that inhibiting β-catenin may be a strategy to improve myocardial fibrosis and cardiac dysfunction after infarction (Qian et al., 2018; Cui et al., 2021; Eid et al., 2021). In addition to the Wnt1/β-catenin pathway, Wnt2 and Wnt4 are involved in myocardial fibrosis. Cardiac Wnt2 and Wnt4 levels were significantly increased on the third day after MI in rats and were consistent with the time of the increase in Col1, Col3 and TGF-β1. The increase in Wnt2 and Wnt4 promoted fibrosis by activating the β-catenin/NF-kB/p65 pathway in a manner dependent on the cooperation of Fzd4/2 and LRP6, while ICG-001 (a β-catenin inhibitor) inhibited this pathway and weakened the fibrosis effect (Yin et al., 2021).

Cardiomyocyte proliferation in the injured myocardium is an important biological process in myocardial repair. Cardiomyocytes are considered terminally differentiated cells with limited regenerative capacity. However, recent studies have demonstrated that nuclear β-catenin has the potential to promote the proliferation of adult cardiomyocytes in a specific time window after MI (Fan et al., 2018; Hauck et al., 2021). An increase in the nuclear import of β-catenin enhanced the proliferation transcriptional activation of related target genes (Axin2, Ccnd1, Myc and Sox2) and induced cardiomyocyte cytokinesis in the infarct and border zones during the 3–10 days after MI, resulting in reduced infarct size and improved cardiac function (Hauck et al., 2021). Furthermore, β-catenin was involved in the differentiation and regeneration of cardiac cells and stem cells. A study showed that the decrease in structural remodeling in β-catenin-knockout mice was the result of enhanced differentiation of resident cardiac progenitor cells, which means that β-catenin downregulation contributes to endogenous cardiac regeneration (Zelarayán et al., 2008). β-catenin inhibition enhances the differentiation of resident c-Kit (+) cells into cardiomyocytes *in vivo* (Hodgkinson et al., 2018). It appears that the effect of β-catenin on the differentiation of cardiac progenitors depends on cell type.

Additionally, angiogenesis in the injured myocardium may be an important biological process in myocardial repair. The newly formed vessels in the infarction area contribute to the transport of oxygen and metabolic substances in new tissues and promote tissue healing. The time of the appearance of β-catenin in the cytoplasm of neovascular endothelial cells within 1 week after infarction was consistent with the formation time of new blood vessels around the infarcted area, indicating that it was involved in the formation of new blood vessels (Blankesteijn et al., 2000). The overexpression of β-catenin enhanced the expression of VEGF and significantly increased the capillary density post MI, which then promoted angiogenesis and tissue healing (Baruah et al., 2017).

### 4.2 β-catenin and arrhythmia

**FIGURE 2:**
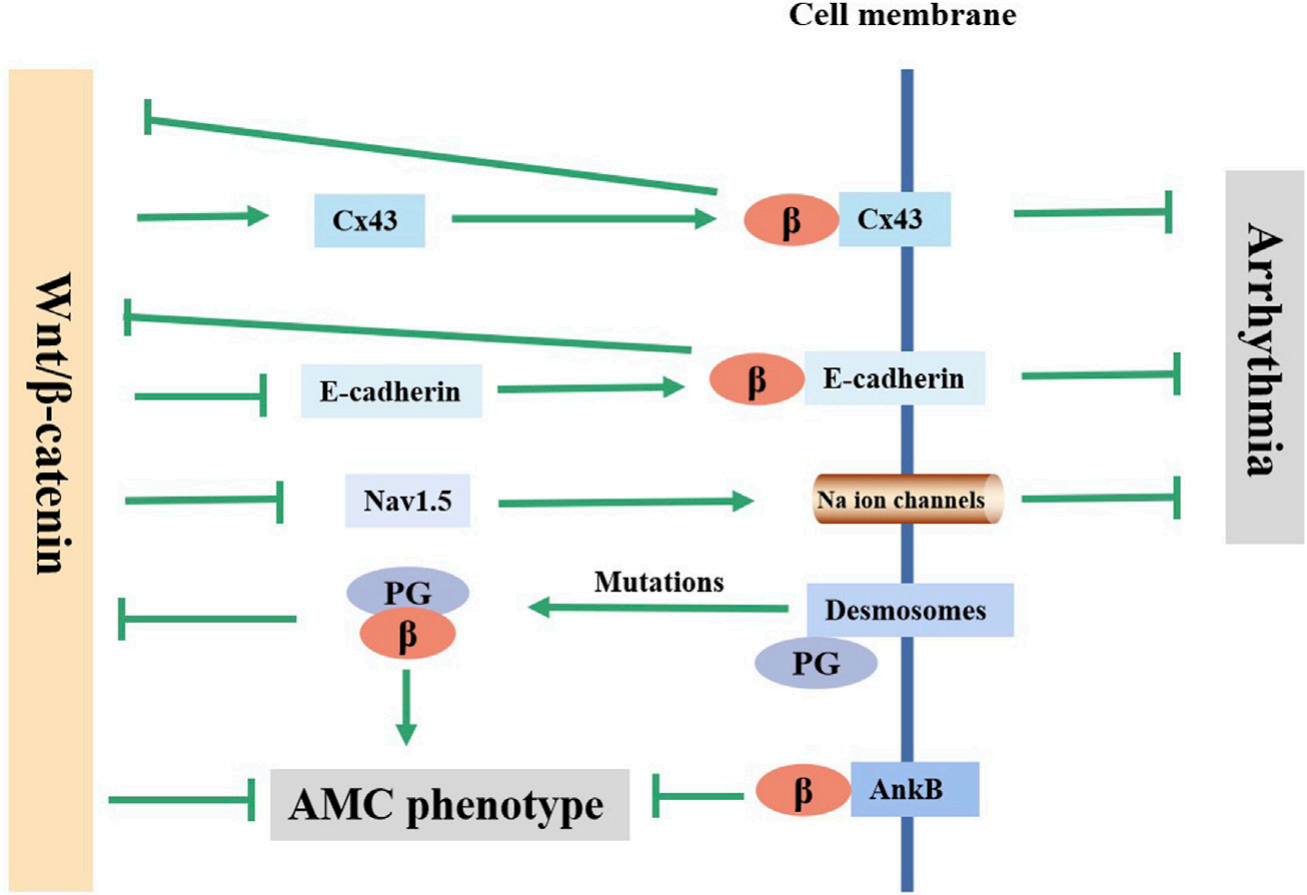
Current knowledge on the regulatory mechanism of Wnt/β-catenin pathway in arrhythmia and ACM. Wnt/β-catenin pathway affects the arrhythmia through regulating the expression of Cx43, E-cadherin and Nav1.5. Mutations in the desmosomal gene can lead to PG free, which inhibits the canonical Wnt/β-catenin pathway and causes AMC phenotype. AnkB inhibits AMC phenotype by co-localizing with β-catenin on the on the membrane.

The pathophysiological basis of arrhythmia involves structural or electrical abnormalities and is mainly caused by acquired factors, such as various cardiovascular diseases such as MI and heart failure (Thomas et al., 2019). Changes in cardiac gap junctions (GJs) after MI are an important factor affecting electrical coupling disorders and conduction abnormalities. Connexin43 (Cx43, gene GJA1) is a major gap junction protein in cardiomyocytes. Changes in the quantity and distribution of Cx43 lead to electrical coupling barriers and conduction anomalies (Hou et al., 2022). A decrease in the expression of Cx43 is closely related to arrhythmia. The Wnt/β-catenin pathway was confirmed to regulate the expression of Cx43 (López et al., 2019). Activation of the Wnt/β-catenin/TCF pathway in neonatal rat cardiomyocytes was confirmed to increase the expression of Cx43 and promote the colocalization of Cx43 and β-catenin in the cell membrane, enhancing intercellular coupling, which in turn negatively regulated the transcriptional activity of β-catenin (Ai et al., 2000; Nakashima et al., 2014). Decreased Cx43 expression, Cx43-containing gap junction remodeling, and conduction abnormalities were observed in mouse cardiac tissue after β-catenin knockout (Swope et al., 2012). These data suggested that β-catenin modulated arrhythmia by interacting with Cx43 (Figure 2). Additionally, the β-catenin/cadherin complex can strengthen the connections between cells. Disruption of the β-catenin/cadherin complex leading to gap instability is one of the causes of arrhythmias (Li et al., 2005).

The Wnt/β-catenin pathway may also affect arrhythmia susceptibility by altering the activity of Na^+^ channels. Voltage-gated cardiac Na^+^ channel activity is associated with cardiac excitation and rapid conduction of action potentials (Turan et al., 2020). Na^+^ channel activity is mainly determined by the Nav1.5 protein (*SCN5A*-encode), the α subunit of the myocardial voltage-gated sodium channel. Rong Huo et al. (Huo et al., 2019) found that enhancing the Wnt/β-catenin/TCF4 pathway could inhibit Nav1.5 protein expression in mice, resulting in increased arrhythmia susceptibility. The possible mechanisms are as follows: 1) After Wnt/β-catenin pathway activation, β-catenin/TCF4 binds to the *Tbx3* (a suppressor of *Scn5a* expression) promoter at -871 bp, directly activates *Tbx3* transcription, and directly inhibits Nav1.5 expression (Gillers et al., 2015; Lu et al., 2020). 2) β-catenin/TCF4 directly binds to the *Scn5a* promoter to inhibit the expression of Nav1.5 (Lu et al., 2020). The inhibition of Nav1.5 by Wnt/β-catenin is responsible for arrhythmia in the acute phase after MI, while the mechanism of arrhythmia in the chronic phase after MI is more likely to involve myocardial remodeling rather than changes in ion channels. Jerry Wang et al. (Wang J. et al., 2021) observed a reduction in the occurrence of arrhythmias in β-catenin-knockout (KO) mice at 8 weeks after MI, which was consistent with reduced scar size and ventricular dilation, and Na^+^/K^+^ channel genes were not different between WT (wild type) and KO mice (Wang J. et al., 2021).

### 4.3 β-catenin and arrhythmogenic cardiomyopathy

Arrhythmic cardiomyopathy (ACM) is an inherited disease characterized by fibrofatty replacement of the ventricular myocardium. Although the incidence is low (from 1/2000 to 1/5000 people worldwide), it can lead to life-threatening arrhythmias and sudden death, especially in young people and athletes, and there is currently no effective treatment for ACM (Stevens et al., 2020). Approximately 85%–90% of ACM-related variants are desmosomal genes, which primarily include plakophilin-2 (*PKP2*), desmoplakin (*DSP*), desmoglein-2 (*DSG2*), democollin-2 (*DSC2*) and plakoglobin (*JUP*) (Gao et al., 2020). Desmosomes are a kind of connection structure between cells. In myocardial tissue, desmosomes, adhesive connections and gap connections constitute intercalated discs (IDs). Many researchers have found that an abnormal Wnt/β-catenin pathway caused by ACM causal mutations is an important mechanism of fibrofatty infiltration.

Mutations in desmosomal genes (such as *DSP*) can cause telomere damage, leading to plakoglobin (PG) dissociation and nuclear localization. PG and β-catenin have a high degree of sequence identity and homology, and they are usually antagonistic on multiple levels (Yin et al., 2022). As a β-catenin competitor, PG could inhibit the typical Wnt/β-catenin-TCF/LEF pathway, increasing the expression of peroxisome proliferation-activated receptor γ (PPARγ), lipogenic transcription factor CCAAT/enhancer binding protein α (C/EBP-α) and its target genes adiponectin and lipoprotein lipase and increasing the levels of procollagen genes Col1a2, Col1a1 and Col3a1, ultimately leading to the human AMC phenotype (Ross et al., 2000; Garcia-Gras et al., 2006; Kim et al., 2013; Cason et al., 2021). In addition, perturbed molecular changes in ID proteins could lead to the inactivation of a component of the IDs: protein kinase C-α (PKC-α). Inactive PKC-α then phosphorylates neurofibromin 2 (NF2) and Yes-associated protein (YAP). The association between p-YAP and p-β-catenin might decrease Wnt/β-catenin signaling pathway activity and enhance adipogenesis (Chen et al., 2014). Furthermore, it was reported that ACM caused by non-telomere protein genes such as *ANK2* (encoding Ankyrin-B, AnkB) was associated with the Wnt/β-catenin signaling pathway. β-catenin and AnkB are molecular chaperones, and AnkB is required for the normal localization of β-catenin (Roberts et al., 2019). The ACM phenotype caused by myocardial-specific knockout of AnkB in mice might be the result of disruption of the auxiliary role of AnkB for the correct localization of β-catenin, which decreased the expression of β-catenin at the IDs. By inhibiting the activity of GSK-3β, the localization of β-catenin in the IDs was partially restored, and the ACM phenotype was improved (Roberts et al., 2019). In addition, SB216763, a typical Wnt signaling activator, could rescue the disease phenotype and restore sodium influx into myocytes in zebrafish ACM models (Asimaki et al., 2014). However, the ACM phenotype following knockout of the murine cardiac *JUP* gene was thought to be associated with an increase in β-catenin/TCF transcriptional activity (Li et al., 2011). In conclusion, dysregulation of the Wnt/β-catenin signaling pathway is a common underlying mechanism of ACM caused by multiple genetic variants, and the regulation of this pathway is a promising therapeutic strategy for ACM (Figure 2).

### 4.4 β-catenin and diabetic cardiomyopathies

Diabetic cardiomyopathy (DCM) is a major cardiovascular complication associated with diabetes that is independent of hypertension, atherosclerosis and other cardiac diseases. DCM is characterized by hypertrophy and myocardial dilatation, as well as abnormal cardiac structure and function, and eventually develops into heart failure. The pathogenesis of DCM remains incompletely understood. It is believed to be related to oxidative stress, inflammation, myocardial apoptosis, autophagy and mitochondrial damage (Avagimyan et al., 2022). Many researchers have confirmed that β-catenin is involved in the development of DCM (Xi et al., 2015; Liu et al., 2020; Chen et al., 2022). In the DCM model induced by streptozotocin (STZ), the protein and mRNA expression levels of Wnt2, β-catenin, and c-Myc were increased in a time-dependent manner, as well as the expression of p-GSK3β (Xi et al., 2015). Similarly, overexpression of Wnt3 and β-catenin was observed in cardiac fibroblasts under high glucose conditions (Hu et al., 2020). Hyperglycemia can cause the overproduction of ROS by the mitochondrial electron-transport chain, which is a common element in DCM-induced injury. Increased oxidative stress products and activation of the Wnt/β-catenin signaling pathway were observed in an STZ-induced DCM mouse model (Liu et al., 2017). Oxidative stress can activate the Wnt/β-catenin pathway, activate the nuclear β-catenin/c-Myc axis and aggravate oxidative stress injury, as we described in Section 4.1.1. After knocking out myocardial β-catenin in the STZ-induced DCM model, diabetic heart dysfunction was improved by inhibiting c-Myc (Liu et al., 2017). Additionally, autophagy is important for maintaining normal cardiac function. STZ-induced activation of the Wnt/β-catenin pathway, increased p-GSK-3β and impaired autophagy were observed in diabetic rats (Wei et al., 2017). Autophagy can be inhibited by mTOR through the phosphorylation of the unc-51-like autophagy activating kinase 1 (ULK1) complex (Wei et al., 2017). GSK-3β inhibits mTOR signaling by phosphorylating tuberous sclerosis (TSC2) (Vigneron et al., 2011). Thus, by inhibiting β-catenin/TCF4/GSK-3β/mTOR, 1,25-dihydroxyvitamin-D3 enhanced autophagy and ameliorated DCM (Wei et al., 2017). In general, β-catenin is related to multiple pathological mechanisms in DCM and is a potential target for the treatment of DCM.

### 4.5 β-catenin and myocardial hypertrophy

Myocardial hypertrophy (MH) is an adaptive change in the myocardium induced by various external stimuli. The markers of MH include myocardial cell volume enlargement and the expression of fetal genes, including atrial natriuretic peptide (ANP), brain natriuretic peptide (BNP), and β-myosin heavy chain (αMHC) (Li et al., 2016). Studies have shown that overexpression of β-catenin can lead to MH (Li et al., 2016; Lin et al., 2021). Overexpression of β-catenin in cardiac myocytes could lead to cell volume increases, actin formation, pathological hypertrophy marker (kinase p38, JNK1/2, ERK1/2, ERK5) activation, and ANP and BNP increases (Lee et al., 2017). Knockout or inhibition of myocardial β-catenin reduced MH (Srivastava et al., 2022). After the slow administration of Ang II in mice, eight Wnt ligands in the heart were upregulated, and the Wnt/β-catenin pathway was activated (Zhao et al., 2018). In Ang-II-induced MH, the expression of β-catenin and c-Myc was upregulated, and the Wnt/β-catenin pathway was activated (Yu et al., 2016; Zhang R. et al., 2022). The Ang-II-mediated hypertrophic response in human ventricular cardiomyocytes includes an increase in inactivated GSK3β (phosphorylation at Ser 9), a decrease in p-β-catenin, and an increase in nuclear β-catenin accumulation (Narasimhan et al., 2019). Moreover, ICG-001 alleviated Ang II-induced myocardial hypertrophy by blocking the Wnt/β-catenin pathway (Zhao et al., 2018). Therefore, the Wnt/β-catenin pathway may play an important role in MH.

## 5 Potential therapeutic inhibitors against Wnt/β-catenin

To date, many researchers have made efforts to develop ideal Wnt/β-catenin pathway inhibitors for the treatment of certain diseases. As a result, many potential therapeutic inhibitors have been developed, including natural and synthetic proteins, antibodies and small molecule compounds. These inhibitors mainly act by inhibiting Wnt or β-catenin activity. At present, some inhibitors have been studied *in vivo* to treat some cardiac diseases. The effects of several Wnt/β-catenin pathway inhibitors on cardiac diseases are shown in Table 2.

**TABLE 2:** Several Wnt/β-catenin pathway inhibitors for cardiac diseases.

Inhibitor	Inhibition of targets	Outcomes	Ref
ICG001	Blocking the β-catenin/CBP protein interaction	Reducing left ventricular wall myocardial hypertrophy and fibrosis in the rat heart with transverse aortic constriction	Methatham et al. (2021)
ICG001		Improving contractile function in chronically infarcted rat myocardium	Sasaki et al. (2013)
IGFBP-4	Binding directly to Wnt or its receptor	Protecting the ischemic heart in mice	Wo et al. (2016)
Cdon	Competing with Wnt for binding to LRP	Inhibiting cardiac fibrosis and cardiomyopathy	Jeong et al. (2017)
Sfrp1	Binding directly to Wnt or its receptor	Effectively protecting aged hearts from AMI injury in aged mice	Tao et al. (2021)
ARNI	Inhibiting β-catenin expression by upregulating sFRP-1	Improving myocardial fibrosis and preventing myocardial remodeling	Liu et al. (2021)
Cardiomogen 1	Inhibiting β-catenin-mediated TCF/LEF-mediated luciferase activity	Increasing newly formed cardiomyocytes and reducing fibrotic scar tissue	Xie et al. (2020)
Wnt-C59	Inhibiting Wnt palmitoylation and secretion	Attenuating pressure overload-induced cardiac hypertrophic	Zhao et al. (2020)
Naringin	Inhibiting β-catenin expression by upregulating GSK3β	Attenuating the inflammatory response after MI	Guo et al. (2022)

## 6 Conclusion remarks

The Wnt/β-catenin signaling pathway is a complex protein network that is involved in various physiological and pathophysiological processes from cardiac development to cardiac muscle remodeling and myocardial repair. Without Wnt, β-catenin does not accumulate in the cytoplasm because there is a destruction complex that typically degrades it. However, under certain circumstances, the Wnt/β-catenin pathway is overactivated, which promotes more β-catenin entry into the nucleus and initiates the expression of downstream target genes (Colozza and Koo, 2021). Recently, abnormal activation of the Wnt/β-catenin signaling pathway has been shown to play an important role in MI, arrhythmias, infarction, ACM, DCM and MH. For example, activation of Wnt/β-catenin signaling can promote oxidative loss, increase the expression of inflammatory factors and delay the repair of myocardial injury after MI, which would further aggravate myocardial injury (Vallée and Lecarpentier, 2018; Liu et al., 2022). These diseases may cause MH and myocardial fibrosis (MF), which is one of the independent risk factors for heart failure (Xiang et al., 2017; Uriel et al., 2018). Although the Wnt/β-catenin pathway has paradoxical roles in myocardial repair and inflammatory responses and even positive roles in arrhythmias and ACM, it is still a disadvantage in most heart diseases.

Therefore, exploring inhibitors against the Wnt/β-catenin signaling pathway is considered a novel method for the treatment of cardiac diseases. The discovered inhibitors act mainly by binding directly to Wnt, inhibiting Wnt palmitoylation and secretion and blocking the β-catenin/CBP protein interaction. These inhibitors have been shown to be effective in treating some cardiac diseases *in vivo*.

In summary, this review provides a detailed summary and analysis of recent studies on the role of β-catenin in cardiac diseases and some potential inhibitors of Wnt/β-catenin. The expression of β-catenin may be a useful biomarker for identifying patients who may best respond to anti-Wnt/β-catenin therapy in cardiac diseases. Even so, the reason that cardiac diseases affect activation of the Wnt/β-catenin signaling pathway and the molecular mechanisms of Wnt/β-catenin involved in cardiac disease progression need to be further studied. Further study and investigation of the regulatory mechanism of the Wnt/β-catenin signaling pathway and the role of inhibitors will provide new ideas for the early diagnosis and precise treatment of related cardiac diseases.
